# Successful treatment of a genetic childhood ataxia due to riboflavin transporter deficiency

**DOI:** 10.1186/s40673-018-0091-0

**Published:** 2018-10-20

**Authors:** Judy Fan, Brent L. Fogel

**Affiliations:** 1 Program in Neurogenetics, Department of Neurology, David Geffen School of Medicine, University of California Los Angeles, Los Angeles, CA 90095 USA; 2 Department of Human Genetics, David Geffen School of Medicine, University of California Los Angeles, 695 Charles E. Young Drive South, Gonda Room 1206, Los Angeles, CA 90095 USA

**Keywords:** Cerebellar Ataxia, Spinocerebellar Ataxia, SLC52A2, Riboflavin, Neurogenetics

## Abstract

**Background:**

Riboflavin transporter deficiency (Brown-Vialetto-Van Laere syndrome) is a rare recessive neurodegenerative disorder that can present with gait ataxia, primarily due to sensory neuropathy as well as cerebellar involvement. Although sensorineural hearing loss, bulbar palsy, and optic atrophy are typical, presentation may be variable and an atypical condition may be difficult to recognize clinically.

**Case presentation:**

Here we report a patient presenting at age 8 with progressive ataxia since the age of 2.5 years with cerebellar atrophy and peripheral polyneuropathy. Whole exome sequencing identified a known pathogenic mutation in the *SLC52A2* gene consistent with a diagnosis of Brown-Vialetto-Van Laere syndrome despite the absence of common symptoms including motor neuropathy, bulbar palsy, optic atrophy, and sensorineural hearing loss. High-dose riboflavin therapy was initiated, symptoms stabilized, metabolic abnormalities resolved, and the patient is doing well with a near-normal examination at age 15.

**Conclusions:**

Riboflavin transporter deficiency can be fatal if left untreated. The excellent outcome of this case illustrates the importance of identifying this potentially treatable neurologic condition. In this patient, clinical diagnosis was limited by an atypical presentation lacking several common features which was overcome through the use of genomic sequencing identifying the pathogenic mutation enabling correct diagnosis and subsequent treatment. Riboflavin transporter deficiency should be considered early in the diagnostic evaluation as a treatable form of ataxia in children, even if patients lack typical features.

**Electronic supplementary material:**

The online version of this article (10.1186/s40673-018-0091-0) contains supplementary material, which is available to authorized users.

## Background

Riboflavin transporter deficiency, also known as Brown-Vialetto-Van Laere syndrome, is a rare autosomal recessive neurodegenerative disorder that can include progressive motor and sensory neuropathy, gait ataxia (typically due to sensory neuropathy), sensorineural hearing loss, bulbar palsy, and optic atrophy [1]. Respiratory insufficiency can lead to ventilator dependence and the outcome can be fatal in childhood [1, 2]. The disease is caused by defects in the human riboflavin transporters RFVT2 and RVFT3, encoded by the *SLC52A2* and *SCL52A3* genes, respectively [1, 2]. Previous reports have noted that response to high-dose riboflavin supplementation can be life-saving and has included stabilization of function, normalization of metabolic abnormalities and audiometry, and improvement in growth, muscle tone, pulmonary function, and visual evoked potentials in some patients [1–4]. Here we report a case of a child originally diagnosed with cerebellar ataxia found to have mutation of *SLC52A2* and successfully treated with high-dose riboflavin.

## Case presentation

The patient was born to consanguineous parents (second cousins) of Lebanese descent. She walked at 18 months, but unsteadily, with toe walking and minimal knee bending. Development was otherwise normal. At the age of 2.5 years, difficulties with balance and coordination were noted and a neurological examination diagnosed her with ataxia. An MRI confirmed cerebellar atrophy, predominantly of the vermis, and she was diagnosed with cerebellar ataxia. At age 4.5 years she was noted to have scoliosis, which was treated with a brace. Genetic testing for common spinocerebellar ataxias, including SCA1, SCA2, SCA3, SCA6, SCA7, and Friedreich ataxia, was negative [5]. Additional single gene testing was performed for rarer forms of spinocerebellar ataxia as well as for Charcot-Marie-Tooth disease and this was also negative. Basic metabolic testing was normal and included levels of lactate, pyruvate, coenzyme-Q10, and fasting glucose.

Symptoms continued to worsen and she was seen for evaluation at our tertiary referral center at age 8 years. Although she experienced regular falls, she was active and able to ride a bike with training wheels. Physical examination revealed appendicular and gait ataxia as well as a multimodal, predominantly sensory, peripheral neuropathy, with areflexia and upgoing toes. Nerve conduction study showed absent sensory responses but normal motor responses in the limbs. Audiology evaluation revealed no sensorineural hearing loss although she was later diagnosed with an auditory processing disorder at 9 years old. There was no other family history of neurological disease. Basic laboratory testing remained normal although very long chain fatty acids showed mildly elevated phytanic acid 3.95 μmol/L (0.37–3.46 μmol/L) and pristanic acid 0.31 μmol/L (≤ 0.28 μmol/L) so a metabolic disorder was considered and genomic sequencing was performed. Whole exome sequencing revealed a homozygous c.916G > A (p.Gly306Arg) variant in the *SLC52A2* gene [6]. Each of her parents was found to be a heterozygous carrier. This is a known pathogenic variant thought to be a founder mutation in the Lebanese population [4].

The patient was started on high-dose riboflavin therapy at 4.5 mg/kg/day at age 9 years. Plasma riboflavin levels ranged between 7.6–27.5 nmol/L (normal 6.2–39.0 nmol/L). Her scale for the assessment and rating of ataxia (SARA) score at presentation was 9.5 (severity range 0–40) and improved to 8 within the first year of treatment. Plasma acylcarnitine profile and urine organic acid levels were monitored throughout treatment and initially showed mild nonspecific abnormalities that subsequently normalized with treatment (see Additional file 1: Table S1). Her riboflavin dose was titrated to a peak of 13 mg/kg/day given the persistent nonspecific elevations seen on metabolic testing but was later reduced to 10.5 mg/kg/day after gastrointestinal upset led to noncompliance and a worsening of symptoms. At age 15 years she is active, participating in sports, and otherwise healthy with a SARA score of 3.0.

## Discussion and conclusions

Riboflavin transporter deficiency is a rare neurodegenerative condition that, if left untreated, can lead to a fatal outcome. A recent review describing 27 patients with RVFT2 deficiency reported that high-dose riboflavin supplementation can often lead to stabilization (8/28, 30%), if not improvement (12/27, 44%), of symptoms [2]. This included benefits in muscle tone, gait, pulmonary function, and fatigability, with normalization of metabolic abnormalities and audiometry [2, 4]. There have also been individual case reports of severe presentations showing remarkable improvement on high-dose riboflavin [7, 8]. The pathophysiology of this condition is still unclear, however, and optimal dosing of riboflavin is unknown [1, 2].

In the majority of patients with riboflavin transporter deficiency, a sensory gait ataxia occurs due to sensory neuropathy [1, 2]. There are, however, descriptions of patients with *SLC52A2* mutations and cerebellar involvement [1, 9]. It may be difficult to distinguish the two in young children. Here we report a patient originally diagnosed with cerebellar atrophy and a corresponding ataxia (likely due to a combination of cerebellar and sensory dysfunction) found to have riboflavin transporter deficiency syndrome caused by mutation of the *SLC52A2* gene although lacking many of the common elements of the classic disorder. Given that the prevalence and expressivity of this disease is currently unknown, atypical or mild cases could be missed in the early evaluation where treatment with riboflavin could be most beneficial. This case serves as an illustration of the importance of recognizing these patients early as a very successful outcome was attained with high-dose riboflavin supplementation over 6 years. Therefore riboflavin transporter deficiency should be considered early in the diagnostic evaluation as a treatable form of ataxia in children, even if patients lack typical features such as motor neuropathy, sensorineural hearing loss, or optic atrophy. Genomic sequencing may be of particular value in these instances as it surveys the entire human genome and, unlike limited gene panels, is unbiased toward disorders with atypical or unusual presentations [6, 10].

## Additional file

Additional file 1: **Table S1.** Laboratory testing. (DOCX 20 kb)
