# Supplementary material for: Successful treatment of a genetic childhood ataxia due to riboflavin transporter deficiency
Source: Cerebellum Ataxias. 2018 Oct 20;5:12. doi: 10.1186/s40673-018-0091-0 (PMC6196015; doi:10.1186/s40673-018-0091-0)
Supplement: Supplementary file 1 — Table S1. Laboratory testing. (DOCX 20 kb) [file 40673_2018_91_MOESM1_ESM.docx]

Additional File 1:

Table S1. Laboratory testing

| **Age** | **SARA score** | **Riboflavin dose**  **(mg/kg/day)** | **Plasma riboflavin**  **(ref 6.2 - 39.0 nmol/L)**  **^(#^ref 5.0 - 50.0 nmol/L)** | **Acylcarnitine profile (umol/L) (ref)** | **Urine organic acids**  **(mmol/mol creatinine) (ref)** |
| --- | --- | --- | --- | --- | --- |
| 9 | 9.5 | 4.5 | 11.9 | Free carnitine: 19.9  (20-70)  C2: 1.93 (2.7-14) | Pimelic: 10 (0-5)  Suberic: 11 (0-8)  Azelaic: 7 (0-10) |
| 10 | 8.0 | 7.4 | 11.8 | Normal | Normal |
| 11 | np | 10.5 | 11.8 | Normal | 3-OH-3-Methylglutaric: 0  (1-13)  Adipic: 0 (1-12) |
| 12 | 5.0 | 13 | 27.5 | C16:1: 0.06 (<0.04) | Isocitric: 18 (22-343)  Citric: 52 (76 – 1309) |
| 13 | 8.0* | 10.5 | 7.6* | Normal | Normal |
| 14 | 4.5 | 10.5 | 15.0^#^ | Normal | Normal |
| 15 | 3.0 | 10.5 | 9.0^#^ | np | np |

* following a period of noncompliance; np, not performed; ref, reference values.
